# Advancing the Analysis of Fatty Acid Composition in Animal-Based Marine Oils Through the Integration of Raman and IR Spectroscopy with Chemometrics

**DOI:** 10.3390/foods15010183

**Published:** 2026-01-05

**Authors:** Fatema Ahmmed, Keith C. Gordon, Asli Card, Daniel P. Killeen, Sara J. Fraser-Miller

**Affiliations:** 1 Te Whai Ao, Dodd-Walls Centre for Photonic and Quantum Technologies and Department of Chemistry, University of Otago, P.O. Box 56, Dunedin 9016, New Zealand; 2 Riddet Institute, Massey University, Private Bag 11222, Palmerston North 4442, New Zealand; 3 The New Zealand Institute for Plant and Food Research Limited, Port Nelson, P.O. Box 5114, Nelson 7043, New Zealanddaniel.killeen@plantandfood.co.nz (D.P.K.); 4 College of Science and Engineering, Flinders University, Bedford Park, Adelaide, SA 5042, Australia; sara.miller@flinders.edu.au

**Keywords:** fish capsule, krill oil, salmon oil, vibrational spectroscopy, data fusion, chemometrics

## Abstract

This study investigated the use of Raman and IR spectroscopy, individually and combined, for quantifying fatty acid methyl ester (FAME) profiles in animal-based marine oils and potential adulterants (palm oil, ω-3 concentrates in ethyl ester, and generic fish oil). FAME profiles are important for assessing oil quality, conventionally determined via gas chromatography. This study aimed to provide a rapid and non-destructive alternative. The study utilized Partial Least Squares Regression (PLSR) alongside Raman (r^2^ = 0.94; RMSEP = 2.4%) and IR spectroscopy (r^2^ = 0.95; RMSEP = 2.3%), demonstrating similar ω-3 fatty acid predictions. Fusion of IR and Raman spectroscopic datasets improved ω-3 fatty acid (r^2^ = 0.96; RMSEP = 1.9%), polyunsaturated fatty acids (PUFA) (r^2^ = 0.83; RMSEP = 4.0%), and saturated fatty acids (SFA) (r^2^ = 0.79; RMSEP = 4.1%) quantification. The study highlights that fusion of IR and Raman spectroscopic datasets presents a promising avenue for non-destructive fatty acid composition assessment.

## 1. Introduction

Fatty acid methyl ester (FAME) profiles are important markers to understand the quality of oil/lipid samples. FAME analysis is typically carried out using gas chromatographic methods that provide accurate and precise results [1,2]. These analyses require a series of sample preparation steps, which require chemicals (such as boron trifluoride, sodium hydroxide) and solvents (such as hexane, methanol, chloroform, and diethyl ether) [3,4]. Vibrational spectroscopy can offer an alternate solvent-free analysis method, which is both rapid and non-destructive [5,6,7,8].

Raman spectroscopy has recently been used to characterize salmon fatty acids, including quantitation of fatty acid unsaturation in intact salmon muscle [9]. Raman spectra of oil extracts can also be used to predict iodine values with a correlation coefficient (r^2^) of 0.87 and a root mean square error of cross-validation (RMSE_CV_) of 2.5 g I_2_/100 g fat using a single PLSR factor [9]. Accurate quantitation of eicosapentaenoic acid (EPA), docosahexenoic acid (DHA), and EPA + DHA has also been reported using Raman spectroscopy with (r^2^ > 0.95, RMSE_cv_ < 2.6%) [7].

Dong et al. (2013) reported a rapid quantification method for predicting fatty acid compositions of edible oil using Raman spectroscopy coupled with least squares support vector machines (LS-SVM) [10]. In that study, high-quality edible oil was mixed with low-price vegetable oils (sunflower seed oil, soybean oil, and corn oil) in proportions ranging from 0% (*V*/*V*) to 100% (*V*/*V*), and a quantitative model predicted α-linolenic acid, linoleic acid, and oleic acid with small error (0.004%, 0.01% and 0.014%, respectively) [10]. Raman spectroscopy was also reported for the quantitative determination of saturated and unsaturated fatty acids composition in pork adipose tissue and melted fat from the same tissue [11]. A partial least squares regression (PLSR) model was used for the prediction of PUFA, MUFA, and SFA with spectra generated directly from adipose tissue, with relatively small error (RMSE_CV_ = 1.5%, 1.5%, and 1.1%, respectively). In fat samples isolated from the same tissues, equivalent PLSR models had lower error than the ‘in-tissue’ measurements for the same measurements (RMSE_CV_ = 1.0%, 1.0%, and 0.6%) [11]. Raman and IR spectroscopy have also been used to analyze the composition of krill oil, including quantifying ω-3 fatty acids and astaxanthin [12].

Despite the widespread application of Raman and IR spectroscopy for the quantitation of fatty acid methyl ester (FAME) profiles, their efficacy in a wide range of marine oil samples, particularly those that include adulterated samples, remains an area of developing interest [13]. This study aims to fill this gap by evaluating the ability of Raman and IR spectroscopy to accurately quantify fatty acid concentrations across a broad range of marine oil samples [14,15]. Another objective of this study is to assess the quality of premium marine oils by detecting and identifying adulterated samples. In order to achieve this, reference FAME data were obtained using gas chromatography-mass spectrometry (GC-MS) and subsequently compared and validated against the data derived from Raman and IR spectroscopic measurements.

## 2. Materials and Methods

### 2.1. Sample Preparations

Six different oil types (krill oil, KO; cod liver oil, CLO; salmon oil, SO; palm oil, PO; ω-3 concentrates in ethyl ester, O3C; and fish oil, FO) were used to prepare a series of oil mixtures (Supplementary Information; Table S1). Briefly, six batches of each valuable oil (KO, CLO, and SO) were used to prepare mixtures with the three adulterants (PO, O3C, and FO). Ethyl ester forms of omega-3 fatty acid concentrates (O3C) and fish oil (FO) were chosen as adulterants due to their cost-effectiveness, availability, and ease of blending with other oils. Four of the six batches for each valuable oil were used to prepare the model set mixtures, and the remaining two batches from each valuable oil type were used to prepare the test set mixtures. PO and FO were selected as adulterants as they are relatively inexpensive. O3C were selected as their sale is prohibited in some markets, e.g., Australia, due to health concerns.

To create a model set, a total of 162 samples were used from four batches of valuable oil (KO1, KO2, KO4, KO6, CLO1, CLO3, CLO4, CLO6, SO2, SO3, SO5, and SO6). These samples were mixed with different weight percentages (ranging from 0 to 50%) of adulterants (PO, O3C, and FO). Additions in these proportions ensured a broad compositional range of fatty acids in the sample set. The remaining two batches of valuable oil (KO3, KO5, CLO2, CLO5, SO1, and SO4), comprising 114 samples, were used to create an independent test set. These samples were also adulterated in the same manner as the model set to validate the results. Table S2 provides the specifics of the compositional mixtures used in both the model and test datasets. The levels of adulteration are variable but often can be up to 50% and can involve the use of palm oil, concentrates, and cheap “fish oil” (sourced from shark, which is what we used in this study) [16,17,18,19,20].

Following the mixing process, the samples were vigorously vortexed and promptly transferred into 4 mL glass vials. These samples were then carefully stored at a temperature of −20 °C until the spectral measurement collection, in order to ensure the stability of the samples and prevent any potential oxidation or structural changes.

### 2.2. FAME Compositions

FAME compositions of the oils were measured as previously described [21]. The proportion of each fatty acid was reported as a percentage of the total integrated fatty acid peak areas for each chromatogram.

### 2.3. Spectral Measurements

#### 2.3.1. Raman Analysis

Raman spectra were obtained in triplicate from each oil sample using a MultiRAM Fourier transform (FT) Raman spectrometer (Bruker Optics, Ettlingen, Germany) equipped with a liquid nitrogen-cooled Ge detector (D418T), and an Nd:YAG continuous wave laser emitting at 1064 nm, and controlled using OPUS 7.5 software as previously described; samples were analyzed in glass vials using a 180° backscattering arrangement. The spectral data were collected across the 4000–200 cm^−1^ range, utilizing a defocused objective with a laser spot size of ~2 mm in diameter, a laser power of 300 mW, a resolution of 4 cm^−1^, and 128 co-added scans per spectrum. The selection of these parameters was based on the procedures outlined in prior studies [6,7,12,21,22].

#### 2.3.2. ATR-Infrared Analysis

The infrared spectra of each oil sample were measured in triplicate using a Vertex 70 spectrometer (Bruker Optics, Ettlingen, Germany) equipped with a GladiATR attenuated total reflectance (ATR) accessory (Pike Technologies, Madison, WI, USA). Each spectrum measurement and IR set-up was similar to the one described previously [12]. Briefly, the spectral information was collected over the spectral window 4000–300 cm^−1^, and background spectra were initially acquired from the cleaned blank ATR crystal before taking measurements of each sample. The resulting spectra were the average of 32 scans at a spectral resolution of 4 cm^−1^.

### 2.4. Spectral Pre-Processing

Spectroscopic data may exhibit baselines arising from non-vibrational spectroscopic signals, such as fluorescence, thermal emission, scattering effects, or variations in the sample’s geometry or condition. To prevent biases caused by these factors, pre-processing techniques such as rubber band baseline correction (RBC) and standard normal variate (SNV) transformation were applied to the Raman spectra over the spectral range of 3100 to 2650 cm^−1^ and 1800 to 660 cm^−1^, using Orange data mining (version 3.40.0, Bioinformatics Laboratory, Faculty of Computer and Information Science, University of Ljubljana, Ljubljana, Slovenia) [23] and The Unscrambler X (version 10.5.1, CAMO, Oslo, Norway), respectively. The IR spectra were pre-processed by performing linear baseline correction (LBC) over the spectral windows of 3050 to 2600 cm^−1^ and 1800 to 300 cm^−1^, followed by SNV transformation in the same spectral region. These pre-processing steps were crucial in removing artefacts caused by baseline curvature, spectral intensity variations, and differences in sample geometry or condition, thereby ensuring accurate and reliable results are obtained from the spectroscopic analyses.

### 2.5. Chemometric Analysis

#### 2.5.1. Principal Component Analysis

Principal Component Analysis (PCA) was utilized to assess the inherent variability within the datasets acquired from GC-MS, Raman, and IR measurements. The major fatty acids (C14:0, C16:0, C18:1n-9, EPA, DPA, DHA, total PUFA, total MUFA, total SFA, total ω-3, and EPA + DPA + DHA) obtained from GC-MS analysis were selected for PCA. For Raman data, PCA was performed on pre-processed datasets within the spectral windows of 3100–2650 cm^−1^ and 1800–660 cm^−1^. Meanwhile, the spectral windows of 3050–2600 cm^−1^ and 1800–300 cm^−1^ were used for the PCA of the IR data. PCA was carried out in The Unscrambler X with the NIPALS algorithm and cross-validated with random cross-validation over 20 segments.

#### 2.5.2. Data Fusion

A low-level fusion approach was employed wherein the pre-processed spectral data from the two different instruments (as described in Section 2.3.1 and Section 2.3.2) were used [12,22,24]. In order to obtain a single matrix, the two spectral datasets were concatenated as described previously [25].

#### 2.5.3. Partial Least Squares Regression

PLSR was carried out for the quantitative analysis of fatty acid composition in oil samples over the spectral ranges similar to PCA of Raman and IR data, as described above. To develop the PLSR models, the pre-processed spectral data obtained from Raman, IR, and the data fused datasets were correlated against the reference FAME values (% of total fatty acids) obtained from GC-MS utilizing the NIPALS algorithm, and were subjected to systematic (112233) cross-validation with the replicated spectra from each sample removed with each fold to minimize bias in the model. The resulting PLSR models were evaluated using an independent test set, and the root mean squared error of prediction (RMSEP) was used as a measure of the model’s efficacy. The performance of the models is presented in Table 1.

## 3. Results and Discussion

### 3.1. Fatty Acid Composition Using GC-MS

Gas chromatography with mass spectrometry (GC-MS) analysis was carried out to identify the fatty acid composition in valuable marine oil (KO, CLO, and SO) and adulterant oil samples (PO, O3C, and FO). Six key fatty acids are presented as % of total fatty acids in Table S3. Fatty acid composition varied significantly across the oil samples. The individual fatty acid content of the valuable oil samples (KO, CLO, and SO) was within the Codex standard of fish oil supplements (Table S3) [26]. These results suggest that none of the marine oils used for this study were adulterated. Palm oil samples are richer in saturated fatty acids, including palmitic acid (C16:0), than the marine oils: krill oil, cod liver oil, and salmon oil samples (Table S3). Tres et al. (2013) reported higher palmitic acid (26–45% of total fatty acids) and oleic acid (37–44%) in various palm oil samples collected from different geographical locations, which is consistent with the palmitic acid (42.4%) and oleic acid (41.8%) content of palm oil used for this work [27]. The absence of long-chain ω-3 fatty acids such as EPA, DPA, and DHA is well known (Table S3) [27,28,29].

KO, CLO, and SO samples were rich in polyunsaturated fatty acids ω-3 fatty acids, including EPA, DPA, and DHA (Table S3), as reported previously [30,31,32]. Xie et al. (2019) previously described the fatty composition of many marine samples, which contained EPA (krill = 14.3–28.0%; anchovy = 5–26%; tuna = 2.5–9%; menhaden =12.5–19%; salmon = 2–11.5%) and DHA (krill = 7.1–15.7%; anchovy =4–26.5%; tuna =21–42.5%; menhaden = 5–11.5%; salmon = 3–14%) at various concentrations [33]. EPA and DHA content in krill oil (EPA = 17.4–20.5%; DHA = 8.0–10.6%) and salmon oil (EPA = 9.9–19.4%; DHA = 9.3–12.2%) in the present study are in agreement with those data.

In addition to inter-species variation [34], fatty acid composition is also prone to seasonal [35] and geographic variation [27], and can be affected by extraction methods [36] and the time of sample harvesting [37,38]. These factors are probably responsible for the variation in the fatty acid content in various marine samples analyzed in the present study. The EPA (8.0–10.1%) and DHA (10.0–11.4%) content in CLO samples obtained in the present study are similar to the Codex value (EPA= 7.0–16.0% and DHA= 6–18%), reported for CLO [26]. However, CLO samples have slightly higher EPA (9.58–15.5%) and DHA (11.9–19.2%) content depending on geographical locations, species, and seasons [39,40,41]. The analysis of FAME data using PCA is given in Figure S1.

### 3.2. Fatty Acid Analysis Using Raman Spectroscopy

**Figure 1:**
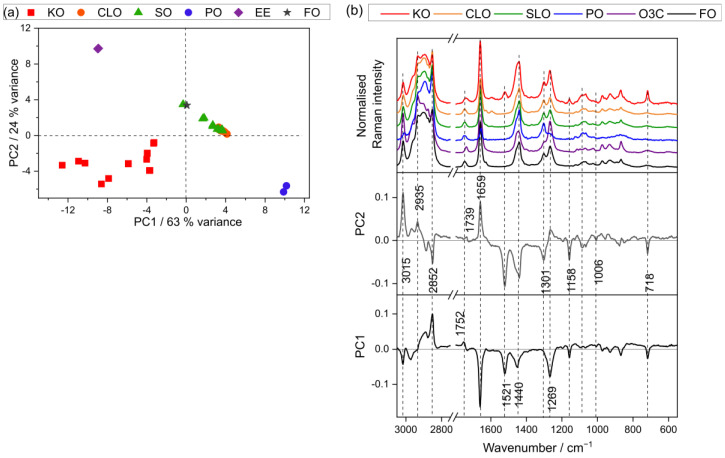
PCA scores and loadings plots of KO, CLO, SO, PO, O3C, and FO samples measured using Raman spectroscopy: (**a**) sample distribution in the PC1 vs. PC2 scores plot (63% and 24% explained variance, respectively), and (**b**) loadings plot with representative spectra of the oil samples.

Spectra from the pure oils (21 batches in total), consisting of KO (6 batches), CLO (6 batches), SO (6 batches), and three cheap oil samples (PO, O3C, and FO), were measured using Raman spectroscopy. The Raman spectra were then analyzed using PCA, which is presented in Figure 1. The first two PCs explain 87% of the total variance between the oil samples (PC1, 63%, and PC2, 24%). The PC scores space shows separation among the different oil samples (Figure 1a).

The first PC separates KO and O3C samples into negative PC1 space, and PO into positive PC1 space. CLO, SO, and FO samples tended to cluster in neutral-to-positive PC1 space (Figure 1a). The spectral variance contributing to this separation is presented in a loading plot (Figure 1b). The major characteristic bands in negative PC1 space correspond to PUFA content (3015, 1669, 1269 cm^−1^, vinyl group), astaxanthin (1521, 1158, 1007 cm^−1^), and other lipid signals (1440, 718 cm^−1^), whereas the positive PC1 is loaded against saturated fatty acids (2852 cm^−1^) and ester signals (1752 cm^−1^).

PC2 tends to separate O3C (positive extreme) from KO and PO (negative extreme) with SLO, FO, and CLO lying between these extremes (Figure 1a). The corresponding PC2 loading plot presented in Figure 1b shows major characteristic lipid bands (3015, 2935, and 1659 cm^−1^) in the positive PC2 portion, indicating a higher proportion of unsaturated fatty acids in O3C than other oil samples (CLO, SO, and FO). The negative PC2 loading portion was associated with dominant astaxanthin bands (1521, 1158, 1007 cm^−1^ in KO samples) and saturated fatty acids bands (2852 cm^−1^ in PO samples) and other lipid signals at 1440, 1301, and 718 cm^−1^.

It is also worthwhile noting that the KO samples had the largest cluster in PC1 vs. PC2 space. This is attributed to the spectral variance in the different compositions of KO samples, in particular astaxanthin, and the sensitivity of Raman spectroscopy towards those particular components.

### 3.3. Fatty Acid Analysis Using Infrared Spectroscopy

**Figure 2:**
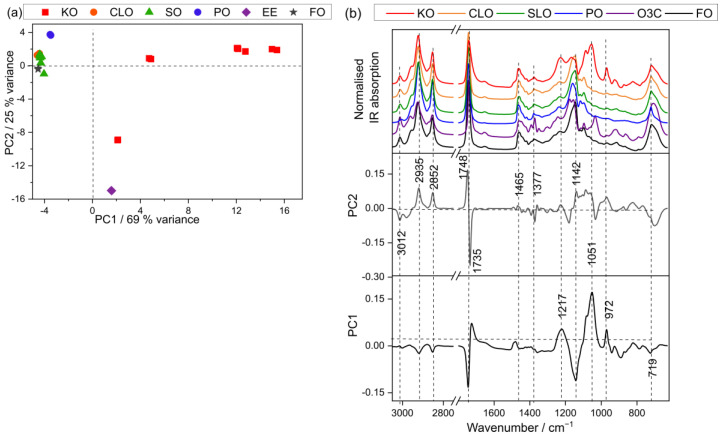
PCA scores plots of KO, CLO, SO, PO, O3C, and FO samples measured using IR. (**a**) PC1 (69% explained variance) versus PC2 (25% explained variance), (**b**) loadings plot with representative spectra of the oil samples.

The IR spectra were obtained from the pure oils (21 batches in total), which consisted of KO (6 batches), CLO (6 batches), salmon oil (6 batches), and three cheap oil samples (PO, O3C, and FO). The IR data were analyzed with PCA, which is presented in Figure 2. 94% of the spectral variance was described by the first two PCs (PC1, 69%, and PC2, 25%).

From the score plot (PC1 vs. PC2) in Figure 2a, it is observed that KO and O3C samples clustered in positive PC1, with KO samples clustering across varied magnitudes of positive PC1 space and O3C in slightly positive PC1 space. The other four oils (CLO, SO, FO, and PO) tended to cluster in negative PC1 space. The spectral variance contributing to this separation is presented in the associated loading plot (Figure 2b). The major characteristic bands in positive PC1 space correspond to lipid signals (1734, 1053, 972 cm^−1^), whereas the negative PC1 loaded against saturated fatty acids (2935, 2852 cm^−1^), ester sifting (1748 cm^−1^), and other lipid signals (1142, 719 cm^−1^). Astaxanthin does not show a strong infrared signal, and thus, variances attributed to it are muted with this technique.

The data from CLO, SO, and FO tended to cluster around the PC2 axis, while KO samples displayed a dispersion across both positive and negative PC2 space. In contrast, PO clustered at the positive end of PC2, and O3C samples clustered at the negative end of PC2.

The corresponding PC2 loading plot, as shown in Figure 2b, revealed prominent bands associated with saturated fatty acids (2935 and 2852 cm^−1^) and ester stretching (1748 and 1142 cm^−1^) in the positive PC2 section. Pure PO exhibited higher band intensities at 2935, 2852, and 1748 cm^−1^, indicating the significant contribution of PO to this separation. Conversely, the negative PC2 loading plot featured bands related to unsaturated fatty acids (3012 cm^−1^), ester signals (1735 cm^−1^), and other lipid-related signals (1377, 1051, and 719 cm^−1^). Pure O3C displayed similar band intensities at 3012, 1735, and 1051 cm^−1^, reaffirming the substantial contribution of O3C to this separation.

The scores separation in the IR data (Figure 2a) differs from that of the Raman (Figure 1a) PCA scores. This suggests that the IR spectral variance reflects other changes in the samples, which may give complementary information to the Raman data for quantifying fatty acids. Therefore, the fusion of IR and Raman data was explored, in addition to the individual techniques, for fatty acid quantification.

### 3.4. Quantitative Analysis of Major Fatty Acid

Partial least squares regression (PLSR) was performed using spectroscopic data and reference FAME data to develop quantitative models for fatty acids using Raman, IR, and the fusion of the two methods. The dataset employed included pure KO, CLO, SO, PO, O3C, and FO, and mixtures thereof for the quantification of major fatty acid content in unknown oil samples. Mixtures were included in this portion of the work to create a dataset that covered a greater range of fatty acid compositions compared to that obtained when using a handful of individual marine oils. Individual PLSR models were developed for total ω-3 fatty acids, total PUFA, EPA + DPA + DHA, EPA (separately), DHA (separately), total MUFA, and total SFA content, and validated using the individual test set samples. The ω-3 fatty acids results are described as a detailed example, and the remaining models are summarized and discussed as a comparison.

### 3.5. Spectroscopic Quantification of ω-3 Fatty Acids

**Figure 3:**
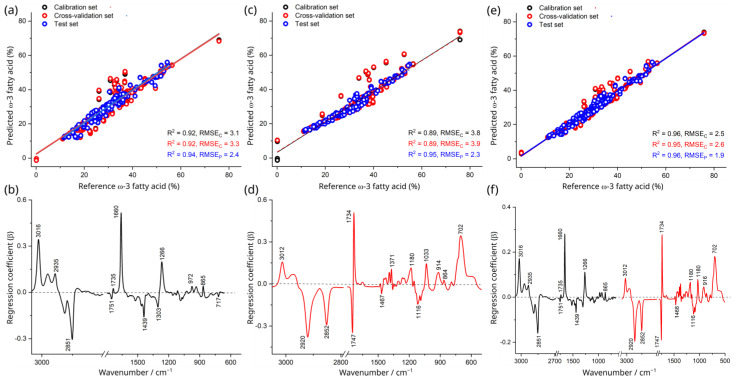
PLSR calibration lines and regression coefficients for quantitative prediction of ω-3 fatty acid % concentration in marine oil (KO, CLO, SO) by Raman (**a**,**b**), IR (**c**,**d**), and low-level fused Raman plus IR data (**e**,**f**). In all cases, two factors were used in the PLSR.

The PLSR model obtained from, Raman, IR and the combined dataset for quantitative measurement of ω-3 fatty acid content in different oil samples yielded a positive correlation between predicted and reference ω-3 fatty acid values with high determination of coefficient (r^2^ = 0.94, r^2^ = 0.95) and low prediction error (RMSE_P_ = 2.4%, RMSE_P_ = 2.3%), respectively (Table S4 and Figure 3a).

The PLSR model obtained from the Raman data revealed that approximately 93% of the total variance in the dataset was explained by the regression coefficient with the first two factors (Figure 3b). The positive regression coefficient associated with the major contribution of unsaturated fatty acids features at 3016 (-C=C-H stretching), 1660 (C=C stretching), and 1266 cm^−1^ (=C-H deformation) (Figure 3b), consistent with what has been reported previously [42]. The negative regression coefficient features shown in Figure 3 were mostly associated with saturated fatty acid signal at 2851 cm^−1^ (C-H stretching) and other characteristic lipid signals at 1439 cm^−1^ (C-H scissoring of methylene group), 1303 cm^−1^ (C-H bending of methylene group), and 717 cm^−1^ (-CH_2_, rocking mode of olefins) [9,42].

The regression coefficient for IR models provided 89% of the total variance in the dataset with the first two factors, as presented in Figure 3d. In the positive regression coefficient, the major bands at 3012 cm^−1^ ((-C=C-H stretching), 1734 cm^−1^ (C=O stretching), 1371 cm^−1^ (-CH_2_ deformation), 1033 cm^−1^ (C-O stretching), and 702 cm^−1^ (-CH_2_ rocking vibration of olefins) in Figure 3d were attributed to characteristic lipid signatures [43,44]. In the negative regression coefficient, other observable spectral features corresponded to saturated compounds at 2920 (-C-H stretching of methylene), 2852 cm^−1^ (-C-H stretching of methylene), 1747 cm^−1^ (C=O stretching of ester groups), and 1116 (C-O stretching of ester), respectively (Figure 3d) [43,45].

The PLSR model obtained from low-level fused data outperformed the single spectroscopic approaches, with better model performance (high r^2^ = 0.96) and higher predictive accuracy (lower RMSE_P_ = 1.9%) compared to Raman (r^2^ = 0.94, RMSE_P_ = 2.4%) and IR (r^2^ = 0.95, RMSE_P_ = 2.3%) models (Table S4). The regression coefficient features derived from the low-level fused data were consistent with individual Raman and IR regression coefficient plots but with slightly different relative peak intensities, as shown in Figure 3f, indicating the combination of spectra could enhance the model performance by integrating the assessed information from different sources (individual techniques) into a single, comprehensive model.

### 3.6. Spectroscopic Quantification of Other Fatty Acids

Calibration models were also developed for the quantification of other major fatty acids, including PUFA, EPA + DPA + DHA, EPA (individually), DHA (individually), MUFA, and SFA content, which is summarized in Table S3. Like the ω-3 model, these models also yielded spectroscopically reasonable regression coefficients, with positive coefficients consistent with the specific fatty acid(s) being quantified. The PLSR model and their corresponding regression coefficients are reported in Supplementary Information (Figures S1–S6).

If we examine the loadings plot, the important positive loadings in the Raman spectra, Figure 3b, include 3016, 1660, and 1266 cm^−1^, associated with vinyl proton stretching, C=C stretching, and bending modes associated with vinyl bonds, respectively [7,22]. Negative loadings at 2851 and 1439 cm^−1^ are associated with C-H stretching of saturated carbohydrates/fatty acids and saturated CH_2_ wagging modes, respectively. The most important loading in the IR spectra is due to the shifted carbonyl feature in the O3C ethyl esters (1760–1710 cm^−1^). This band is far more intense in the IR spectra than the Raman, highlighting the complementarity of the two techniques and illustrating why data fusion is appropriate for detecting adulteration with different fatty acids (FO, SO) and different fatty acid chemical formats (O3C).

In this dataset, three different outcomes were observed with respect to the performance of data fusion versus the individual techniques. These were as follows:

1. The fused model outperformed both individual techniques, indicating that complementary information was provided by each technique to build a better model.
2. The fused model gave a similar performance to the best-performing technique, indicating no additional advantage with the inclusion of the second technique.
3. The fused model performance was intermediate to the individual techniques, indicating the model is effectively an average of the two models’ performance.

When analyzing various fatty acids, the quantification methods exhibited differing levels of accuracy. EPA was most effectively measured using infrared (IR) and low-level data fusion techniques, showing a strong correlation (r^2^ = 0.95) and minimal error (RMSE_P_ = 1.5%). Raman spectroscopy, while slightly less accurate (r^2^ = 0.86, RMSE_P_ = 2.4%), still performed reasonably well for EPA content determination. Fused data performed at the intermediate level to the best individual technique, which could be an example of situation 3.

In the combined assessment of EPA, DPA, and DHA, low-level data fusion and IR demonstrated superiority over Raman. Low-level fused data (r^2^ = 0.95, RMSE_P_ = 2.1%) and IR models (r^2^ = 0.95, RMSE_P_ = 2.1%) improved model performance compared to the Raman model (r^2^ = 0.90, RMSE_P_ = 2.9%), which could be an example of situation 2. In contrast, Raman showed improvement in quantifying PUFA compared to IR. This was another example of situation 2—fused data performed at a similar level to the best individual technique. For the assessment of fatty acid composition, the low-level fused model performed similarly to the IR model (r^2^ = 0.75, RMSEP = 5.2%) but better than the Raman model (r^2^ = 0.66, RMSE_P_ = 6.1%). In the assessment of SFA content, the PLSR model derived from low-level fused data yielded slightly better prediction (r^2^ = 0.79, RMSE_P_ = 4.1%) than the individual Raman technique (r^2^ = 0.71, RMSE_P_ = 4.8%) and the IR technique (r^2^ = 0.78, RMSE_P_ = 4.3%). In the determination of MUFA, the low-level fused model performed similarly to the IR model (r^2^ = 0.75, RMSE_P_ = 5.2%) but better than the Raman model (r^2^ = 0.66, RMSE_P_ = 6.1%).

## 4. Conclusions

As global supply chains intermingle and adulteration of ‘premium’ products becomes more prevalent, there is a growing need for rapid, non-destructive methods for the detection of nefarious activities. This study suggests that Raman and IR spectroscopy, as well as data fusion strategies, in combination with PLSR, may offer a method for rapid determination of major fatty acid composition in a wide range of commercial marine oil samples. Raman (r^2^ = 0.94; RMSE_P_ = 2.4%) and IR (r^2^ = 0.95; RMSE_P_ = 2.3%) showed comparable results for predicting ω-3 fatty acids, whereas low-level fusion significantly improved the model performance and predictive accuracy for quantifying ω-3 fatty acids (r^2^ =0.96; RMSE_P_ = 1.9%), PUFA (r^2^ =0.83; RMSE_P_ = 4.0%), and SFA (r^2^ =0.79; RMSE_P_ = 4.1%) content. IR and low-level fusion also produce comparable prediction results for EPA + DPA + DHA, DHA (individually), and MUFA quantification. The EPA and DHA are almost similar in structure and provide spectral homology, which makes it difficult to quantify them independently in oils containing both compounds. In order to solve this problem, data fusion of IR and Raman data could be a viable way to overcome this analytical challenge. This offer means of detecting adulteration as both infrared and Raman instruments are available in very portable hand-held configurations. Thus, it may be possible to evaluate high-value animal marine oils, such as krill oil, at many points in the production-to-consumer chain.

## Figures and Tables

**Table 1:** The model performance of PLSR for quantification of major fatty acids (FAs) content in krill oil, cod liver oil, and salmon oil. The italicized rows indicate the best-performing model for each FAs type.

		No. Factors	Calibration		Cross-Validation		Prediction (Test Set)			
			r^2^	RMSE_C_	r^2^	RMSE_cv_	r^2^	Slope	Offset	RMSE_P_
**Total ω-3% (model range: 0 to 100%)**	Raman	2	0.92	3.1%	0.92	3.3%	0.94	0.97	1.1	2.4%
	IR	2	0.89	3.8%	0.89	3.9%	0.95	0.88	3.4	2.3%
	Low-level fusion	*2*	*0.96*	*2.5%*	*0.95*	*2.6%*	*0.96*	*0.98*	*0.56*	*1.9%*
**EPA%** **(model range: 0 to 100%)**	Raman	2	0.91	2.1%	0.90	2.2%	0.86	0.84	2.4	2.4%
	IR	*2*	*0.91*	*2.2%*	*0.90*	*2.2%*	*0.95*	*0.86*	*2.2*	*1.5%*
	Low-level fusion	2	0.95	1.6%	0.95	1.6%	0.94	0.90	1.6	1.7%
**DHA%** **(model range: 0 to 100%)**	Raman	2	0.86	1.5%	0.85	1.6%	0.83	0.83	2.2	1.5%
	IR	*2*	*0.80*	*1.8%*	*0.78*	*1.8%*	*0.90*	*0.78*	*2.0*	*1.1%*
	Low-level fusion	*2*	*0.91*	*1.2%*	*0.91*	*1.2%*	*0.91*	*0.86*	*1.4*	*1.1%*
**EPA + DPA + DHA%** **(model range: 0 to 100%)**	Raman	2	0.92	3.2%	0.91	3.3%	0.90	0.95	1.9	2.9%
	IR	*2*	*0.89*	*3.7%*	*0.89*	*3.8%*	*0.95*	*0.86*	*3.6*	*2.1%*
	Low-level fusion	*2*	*0.95*	*2.5%*	*0.95*	*2.6%*	*0.95*	*0.95*	*1.3*	*2.1%*
**Total PUFA%** **(model range: 0 to 100%)**	Raman	*2*	*0.90*	*3.5%*	*0.89*	*3.6%*	*0.83*	*0.85*	*4.1*	*4.0%*
	IR	2	0.88	3.7%	0.88	3.8%	0.79	0.76	6.7	4.4%
	Low-level fusion	*2*	*0.94*	*2.7%*	*0.94*	*2.8%*	*0.83*	*0.86*	*3.6*	*4.0%*
**MUFA%** **(model range: 0 to 100%)**	Raman	2	0.67	6.2%	0.66	6.3%	0.66	0.59	17.3	6.1%
	IR	*2*	*0.74*	*5.6%*	*0.74*	*5.7%*	*0.75*	*0.84*	*5.6*	*5.2%*
	Low-level fusion	*2*	*0.74*	*5.5%*	*0.74*	*5.6%*	*0.76*	*0.80*	*7.7*	*5.2%*
**SFA%** **(model range: 0 to 100%)**	Raman	3	0.72	4.4%	0.71	4.5%	0.71	0.56	11.6	4.8%
	IR	3	0.73	4.2%	0.72	4.3%	0.78	0.74	8.4	4.3%
	Low-level fusion	*3*	*0.74*	*4.1%*	*0.72*	*4.2%*	*0.79*	*0.70*	*8.8*	*4.1%*

## Data Availability

The data presented in this study are available on request from the corresponding author due to university policy.

## Supplementary Materials

The following supporting information can be downloaded at https://www.mdpi.com/article/10.3390/foods15010183/s1, Table S1: A summary of the studied samples with sample name, country of origin, company, batch number, and best before date; Table S2: Summary of the sample mixtures used in this study. Samples are expressed as weight percentages (% *w*/*w*). Abbreviations: VO = valuable oil; K1 to K6 = Krill oil batch; CLO1 to CLO6 = cod liver oil batch; SO1 to SO6 = salmon oil batch; PO = palm oil; O3C = ω-3 concentrates in ethyl ester; FO = fish oil; M = model set; T = test set; Table S3: Fatty acid composition (% of total fatty acids) in krill oil (KO), cod liver oil (CLO), salmon oil (SO), palm oil (PO), ω-3 concentrates in ethyl ester (O3C) and fish oil (FO) from chromatographic analysis; Figure S1: PCA of FAME profiles of six oils: scores (**a**) and loading plots (**b**) for three valuable oils (KO, CLO, SO: each with six different batches) and three cheap oils (PO, O3C, FO); Figure S2: PLSR calibration lines and regression coefficients for quantitative prediction of EPA% concentration in marine oil (KO, CLO, SO) by Raman (**a**,**b**), IR (**c**,**d**) and low-level fused Raman plus IR data (**e**,**f**); Figure S3: PLSR calibration lines and regression coefficients for quantitative prediction of DHA% concentration in marine oil (KO, CLO, SO) by Raman (**a**,**b**), IR (**c**,**d**) and low-level fused Raman plus IR data (**e**,**f**); Figure S4: PLSR calibration lines and regression coefficients for quantitative prediction of EPA + DPA + DHA % concentration in marine oil (KO, CLO, SO) by Raman (**a**,**b**), IR (**c**,**d**) and low-level fused Raman plus IR data (**e**,**f**); Figure S5: PLSR calibration lines and regression coefficients for quantitative prediction of PUFA % concentration in marine oil (KO, CLO, SO) by Raman (**a**,**b**), IR (**c**,**d**) and low-level fused Raman plus IR data (**e**,**f**); Figure S6: PLSR calibration lines and regression coefficients for quantitative prediction of MUFA % concentration in marine oil (KO, CLO, SO) by Raman (**a**,**b**), IR (**c**,**d**) and low-level fused Raman plus IR data (**e**,**f**); Figure S7: PLSR calibration lines and regression coefficients for quantitative prediction of SFA % concentration in marine oil (KO, CLO, SO) by Raman (**a**,**b**), IR (**c**,**d**) and low-level fused Raman plus IR data (**e**,**f**).
