# Supplementary material for: Advancing the Analysis of Fatty Acid Composition in Animal-Based Marine Oils Through the Integration of Raman and IR Spectroscopy with Chemometrics
Source: Foods. 2026 Jan 5;15(1):183. doi: 10.3390/foods15010183 (PMC12785698; doi:10.3390/foods15010183)
Supplement: Supplementary file 1 [file foods-15-00183-s001.zip › foods-4028844-supplementary.pdf]

Supporting information for

Advancing the analysis of fatty acid composition in valuable marine oils through the integration of Raman and IR spectroscopy with chemometrics

Fatema Ahmmed[1,2], Keith C. Gordon[1,2*] Asli Card[3], Daniel P. Killeen[3] and Sara J. Fraser-Miller[4]

1 Te Whai Ao, Dodd-Walls Centre for Photonic and Quantum Technologies and Department of Chemistry, University of Otago, P.O. Box 56, Dunedin 9016, New Zealand.
2 Riddet Institute, Massey University, Private Bag 11222, Palmerston North 4442, New Zealand.
3 The New Zealand Institute for Plant and Food Research Limited, Box 5114, Port Nelson, Nelson 7043, New Zealand
4 College of Science and Engineering, Flinders University, Bedford Park, SA 5042, Australia
* Correspondence: keith.gordon@otago.ac.nz

Table S1: A summary of the studied samples with sample name, country of origin and best before date; Table S2. Summary of the sample mixtures used in this study. Samples are expressed as weight percentages (% w/w). Abbreviations: VO = valuable oil; K1 to K6 = Krill oil batch; CLO1 to CLO6 = cod liver oil batch; SO1 to SO6 = salmon oil batch; PO = palm oil; O3C = ω-3 concentrates in ethyl ester; FO = Fish oil; M = model set; T = test set; Table S3: Fatty acid composition (% of total fatty acids) in krill oil (KO), cod liver oil (CLO), salmon oil (SO), palm oil (PO), ω-3 concentrates in ethyl ester (O3C) and fish oil (FO); Table S4: The model performance of PLSR for quantification of major fatty acids (FAs) content in krill oil, cod-liver oil and salmon oil. The *italicized* rows indicate the best performing model for each FAs type; Figure S 1: An overview of all oil samples used in this experiment. Abbreviation: KO = krill oil; CLO= cod liver oil; SO=salmon oil; PO = palm oil; O3C = ethyl ester; FO = fish oil; Figure S2: PLSR calibration lines and regression coefficients for quantitative prediction of EPA% concentration in marine oil (KO, CLO, SO) by Raman (a,b), IR (c,d) and low-level fused Raman plus IR data (e,f); Figure S3: PLSR calibration lines and regression coefficients for quantitative prediction of DHA% concentration in marine oil (KO, CLO, SO) by Raman (a,b), IR (c,d) and low-level fused Raman plus IR data (e,f); Figure S4: PLSR calibration lines and regression coefficients for quantitative prediction of EPA+DPA+DHA % concentration in marine oil (KO, CLO, SO) by Raman (a,b), IR (c,d) and low-level fused Raman plus IR data (e,f); Figure S5: PLSR calibration lines and regression coefficients for quantitative prediction of PUFA % concentration in marine oil (KO, CLO, SO) by Raman (a,b), IR (c,d) and low-level fused Raman plus IR data (e,f); Figure S6: PLSR calibration lines and regression coefficients for quantitative prediction of MUFA % concentration in marine oil (KO, CLO, SO) by Raman (a,b), IR (c,d) and low-level fused Raman plus IR data (e,f); Figure S7: PLSR calibration lines and regression coefficients for quantitative prediction of SFA % concentration in marine oil (KO, CLO, SO) by Raman (a,b), IR (c,d) and low-level fused Raman plus IR data (e,f)

Table S2: A summary of the studied samples with sample name, country of origin, company, batch number and best before date

Sample name	Country of origin	best before date
**Antarctic krill oil (K1)**	New Zealand	07/2023
**Krill oil (K2)**	New Zealand	03/2023
**Krill oil (K3)**	New Zealand	01/2023
**Krill oil (K4)**	New Zealand	08/2023
**Krill oil (K5)**	USA	06/2023
**Krill oil (K6)**	New Zealand	5/2024
**Cod liver oil (CLO1)**	New Zealand	11/2022
**Cod liver oil (CLO2)**	Australia	11/2024
**Cod liver oil (CLO3)**	USA	02/2023`
**Cod liver oil (CLO4)**	Norway	05/2025
**Cod liver oil (CLO5)**	Norway	03/2025
**Cod liver oil (CLO6)**	Norway	22/2024
**Salmon oil (SO1)**	USA	086024
**Salmon oil (SO2)**	Canada	01/2024
**Salmon oil (SO3)**	USA	02/2023
**Salmon oil (SO4)**	Norway	01/2024
**ω -3 salmon oil (SO5)**	USA	11/22

**Salmon oil (SO6)**	USA	05/24
**Fish oil (**O3C**)**	New Zealand	09/2021
**Palm oil (PO)**	New Zealand	12/2022
**Fish oil (FO)**	New Zealand	07/2023

Table S2. Summary of the sample mixtures used in this study. Samples are expressed as weight percentages (% w/w). Abbreviations: VO = valuable oil; K1 to K6 = Krill oil batch; CLO1 to CLO6 = cod liver oil batch; SO1 to SO6 = salmon oil batch; PO = palm oil; O3C = ω-3 concentrates in ethyl ester; FO = Fish oil; M = model set; T = test set.

Predominant oil type	Batch	VO (% w/w)	PO (% w/w)	O3C (% w/w)	FO (% w/w)	Dataset (M or T)
	KO1	100.0	0.0	0.0	0.0	M
	KO1	97.8	0.0	0.0	2.2	M
	KO1	89.8	0.0	0.0	10.2	M
	KO1	70.0	0.0	0.0	30.0	M
	KO1	50.2	0.0	0.0	49.8	M
	KO1	94.9	0.0	5.1	0.0	M
	KO1	79.6	0.0	20.4	0.0	M
	KO1	97.8	2.2	0.0	0.0	M
	KO1	89.2	10.8	0.0	0.0	M
	KO1	69.6	30.4	0.0	0.0	M
	KO1	51.6	48.4	0.0	0.0	M
	KO1	60.0	0.0	40.0	0.0	M
	KO1	79.9	0.0	20.1	0.0	M
	KO2	100.0	0.0	0.0	0.0	M
	KO2	94.5	0.0	0.0	5.5	M
	KO2	94.4	0.0	0.0	5.6	M
	KO2	90.8	0.0	0.0	9.2	M
	KO2	90.3	0.0	0.0	9.7	M
	KO2	80.4	0.0	0.0	19.6	M
	KO2	59.7	0.0	0.0	40.3	M
	KO2	97.4	0.0	2.6	0.0	M
	KO2	89.8	0.0	10.2	0.0	M
	KO2	70.6	0.0	29.4	0.0	M
	KO2	50.1	0.0	49.9	0.0	M

**Krill oil samples**	KO2	50.0	0.0	50.0	0.0	M
	KO2	94.7	5.3	0.0	0.0	M
	KO2	79.6	20.4	0.0	0.0	M
	KO2	75.8	24.2	0.0	0.0	M
	KO2	75.0	25.0	0.0	0.0	M
	KO2	60.7	39.3	0.0	0.0	M
	KO2	59.9	40.1	0.0	0.0	M
	KO4	100.0	0.0	0.0	0.0	M
	KO4	98.2	0.0	0.0	1.8	M
	KO4	89.6	0.0	0.0	10.4	M
	KO4	70.7	0.0	0.0	29.4	M
	KO4	49.8	0.0	0.0	50.2	M
	KO4	93.6	0.0	6.4	0.0	M
	KO4	80.1	0.0	19.9	0.0	M
	KO4	60.6	0.0	39.4	0.0	M
	KO4	98.0	2.0	0.0	0.0	M
	KO4	90.3	9.7	0.0	0.0	M
	KO4	70.4	29.6	0.0	0.0	M
	KO4	49.7	50.3	0.0	0.0	M
	KO6	100.0	0.0	0.0	0.0	M
	KO6	95.5	0.0	0.0	4.5	M
	KO6	79.9	0.0	0.0	20.1	M
	KO6	59.9	0.0	0.0	40.1	M
	KO6	98.0	0.0	2.0	0.0	M
	KO6	89.9	0.0	10.1	0.0	M
	KO6	70.9	0.0	29.1	0.0	M
	KO6	50.3	0.0	49.7	0.0	M
	KO6	94.4	5.6	0.0	0.0	M
	KO6	79.9	20.1	0.0	0.0	M
	KO6	60.1	39.9	0.0	0.0	M
	KO6	80.2	19.8	0.0	0.0	M

	KO6	79.8	0.0	0.0	20.2	M
	KO6	90.3	0.0	9.7	0.0	M
	KO6	94.7	5.3	0.0	0.0	M
	KO6	94.8	0.0	0.0	5.2	M
	KO6	99.1	0.0	0.9	0.0	M
	KO3	100.0	0.0	0.0	0.0	T
	KO3	95.5	0.0	0.0	4.5	T
	KO3	83.9	0.0	0.0	16.1	T
	KO3	79.7	0.0	0.0	20.3	T
	KO3	79.2	0.0	0.0	20.8	T
	KO3	74.8	0.0	0.0	25.2	T
	KO3	54.1	0.0	0.0	45.9	T
	KO3	50.0	0.0	0.0	50.0	T
	KO3	95.6	0.0	4.4	0.0	T
	KO3	90.8	0.0	9.2	0.0	T
	KO3	74.5	0.0	25.5	0.0	T
	KO3	70.1	0.0	29.9	0.0	T
	KO3	69.8	0.0	30.2	0.0	T
	KO3	64.7	0.0	35.3	0.0	T
	KO3	50.2	0.0	49.8	0.0	T
	KO3	84.5	15.5	0.0	0.0	T
	KO3	74.9	25.1	0.0	0.0	T
	KO3	54.1	45.9	0.0	0.0	T
	KO3	49.5	50.5	0.0	0.0	T
	KO3	50.0	0.0	0.0	50.0	T
	KO5	100.0	0.0	0.0	0.0	T
	KO5	98.2	0.0	0.0	1.8	T
	KO5	92.1	0.0	0.0	7.9	T
	KO5	75.1	0.0	0.0	24.9	T
	KO5	64.9	0.0	0.0	35.1	T
	KO5	50.4	0.0	0.0	49.6	T

	KO5	98.3	0.0	1.7	0.0	T
	KO5	85.0	0.0	15.0	0.0	T
	KO5	75.6	0.0	24.4	0.0	T
	KO5	55.3	0.0	44.7	0.0	T
	KO5	49.7	0.0	50.3	0.0	T
	KO5	98.5	1.5	0.0	0.0	T
	KO5	92.4	7.6	0.0	0.0	T
	KO5	75.2	24.9	0.0	0.0	T
	KO5	65.3	34.7	0.0	0.0	T
	KO5	49.8	50.2	0.0	0.0	T
	KO5	92.5	0.0	0.0	7.5	T
	KO5	98.8	0.0	0.0	1.2	T
	CLO3	100.0	0.0	0.0	0.0	M
	CLO3	93.7	0.0	0.0	6.3	M
	CLO3	79.0	0.0	0.0	21.0	M
	CLO3	59.6	0.0	0.0	40.4	M
	CLO3	97.3	0.0	2.7	0.0	M
	CLO3	89.7	0.0	10.3	0.0	M
	CLO3	69.1	0.0	30.9	0.0	M
	CLO3	50.0	0.0	50.0	0.0	M
	CLO3	93.8	6.2	0.0	0.0	M
	CLO3	79.3	20.7	0.0	0.0	M
	CLO3	59.9	40.1	0.0	0.0	M
	CLO4	100.0	0.0	0.0	0.0	M
	CLO4	97.6	0.0	0.0	2.4	M
	CLO4	89.8	0.0	0.0	10.2	M
	CLO4	69.6	0.0	0.0	30.4	M
	CLO4	49.6	0.0	0.0	50.4	M
	CLO4	94.5	0.0	5.5	0.0	M
	CLO4	79.9	0.0	20.1	0.0	M

**Cod liver oil samples**	CLO4	60.0	0.0	40.0	0.0	M
	CLO4	97.2	2.8	0.0	0.0	M
	CLO4	89.4	10.6	0.0	0.0	M
	CLO4	70.3	29.7	0.0	0.0	M
	CLO4	49.5	50.5	0.0	0.0	M
	CLO6	100.0	0.0	0.0	0.0	M
	CLO6	94.2	0.0	0.0	5.8	M
	CLO6	79.3	0.0	0.0	20.7	M
	CLO6	60.0	0.0	0.0	40.0	M
	CLO6	99.1	0.0	0.9	0.0	M
	CLO6	89.6	0.0	10.4	0.0	M
	CLO6	69.8	0.0	30.2	0.0	M
	CLO6	50.6	0.0	49.4	0.0	M
	CLO6	93.7	6.3	0.0	0.0	M
	CLO6	79.8	20.2	0.0	0.0	M
	CLO6	59.5	40.5	0.0	0.0	M
	CLO2	100.0	0.0	0.0	0.0	T
	CLO2	98.4	0.0	0.0	1.6	T
	CLO2	89.6	0.0	0.0	10.4	T
	CLO2	74.6	0.0	0.0	25.4	T
	CLO2	57.6	0.0	0.0	42.4	T
	CLO2	50.4	0.0	0.0	49.6	T
	CLO2	98.1	0.0	1.9	0.0	T
	CLO2	91.8	0.0	8.2	0.0	T
	CLO2	74.9	0.0	25.1	0.0	T
	CLO2	65.4	0.0	34.6	0.0	T
	CLO2	49.7	0.0	50.3	0.0	T
	CLO2	97.6	2.4	0.0	0.0	T
	CLO2	88.9	11.1	0.0	0.0	T
	CLO2	74.7	25.3	0.0	0.0	T
	CLO2	57.1	42.9	0.0	0.0	T

	CLO2	50.9	49.2	0.0	0.0	T
	CLO5	100.0	0.0	0.0	0.0	T
	CLO5	97.6	0.0	0.0	2.4	T
	CLO5	91.8	0.0	0.0	8.2	T
	CLO5	74.2	0.0	0.0	25.8	T
	CLO5	64.5	0.0	0.0	35.5	T
	CLO5	52.0	0.0	0.0	48.0	T
	CLO5	98.6	0.0	1.4	0.0	T
	CLO5	89.3	0.0	10.7	0.0	T
	CLO5	74.6	0.0	25.4	0.0	T
	CLO5	57.2	0.0	42.8	0.0	T
	CLO5	49.5	0.0	50.5	0.0	T
	CLO5	97.9	2.1	0.0	0.0	T
	CLO5	91.9	8.1	0.0	0.0	T
	CLO5	74.9	25.1	0.0	0.0	T
	CLO5	64.1	35.9	0.0	0.0	T
	CLO5	50.0	50.0	0.0	0.0	T
	SO2	100.0	0.0	0.0	0.0	M
	SO2	95.1	0.0	0.0	4.9	M
	SO2	80.1	0.0	0.0	19.9	M
	SO2	60.1	0.0	0.0	39.9	M
	SO2	94.3	0.0	5.7	0.0	M
	SO2	80.0	0.0	20.0	0.0	M
	SO2	60.0	0.0	40.0	0.0	M
	SO2	99.0	1.0	0.0	0.0	M
	SO2	90.1	9.9	0.0	0.0	M
	SO2	70.3	29.7	0.0	0.0	M
	SO2	50.0	50.0	0.0	0.0	M
	SO3	100.0	0.0	0.0	0.0	M
	SO3	98.5	0.0	0.0	1.5	M

Salmon oil samples	SO3	90.1	0.0	0.0	9.9	M
	SO3	70.1	0.0	0.0	29.9	M
	SO3	49.8	0.0	0.0	50.2	M
	SO3	98.9	0.0	1.1	0.0	M
	SO3	89.8	0.0	10.2	0.0	M
	SO3	70.3	0.0	29.7	0.0	M
	SO3	50.3	0.0	49.7	0.0	M
	SO3	95.0	5.0	0.0	0.0	M
	SO3	79.8	20.2	0.0	0.0	M
	SO3	60.0	40.0	0.0	0.0	M
	SO5	100.0	0.0	0.0	0.0	M
	SO5	95.2	0.0	0.0	4.8	M
	SO5	80.2	0.0	0.0	19.8	M
	SO5	59.9	0.0	0.0	40.1	M
	SO5	95.0	0.0	5.0	0.0	M
	SO5	80.0	0.0	20.0	0.0	M
	SO5	60.0	0.0	40.0	0.0	M
	SO5	98.9	1.1	0.0	0.0	M
	SO5	89.5	10.5	0.0	0.0	M
	SO5	70.3	29.7	0.0	0.0	M
	SO5	50.0	50.0	0.0	0.0	M
	SO6	100.0	0.0	0.0	0.0	M
	SO6	98.8	0.0	0.0	1.2	M
	SO6	89.8	0.0	0.0	10.2	M
	SO6	69.7	0.0	0.0	30.3	M
	SO6	50.3	0.0	0.0	49.7	M
	SO6	99.1	0.0	0.9	0.0	M
	SO6	89.8	0.0	10.2	0.0	M
	SO6	69.9	0.0	30.1	0.0	M
	SO6	50.0	0.0	50.0	0.0	M
	SO6	94.1	5.9	0.0	0.0	M

	SO6	80.2	19.8	0.0	0.0	M
	SO6	60.1	39.9	0.0	0.0	M
	SO1	100.0	0.0	0.0	0.0	T
	SO1	98.8	0.0	0.0	1.2	T
	SO1	92.6	0.0	0.0	7.4	T
	SO1	74.6	0.0	0.0	25.4	T
	SO1	64.6	0.0	0.0	35.4	T
	SO1	49.9	0.0	0.0	50.1	T
	SO1	98.8	0.0	1.2	0.0	T
	SO1	84.7	0.0	15.3	0.0	T
	SO1	57.1	0.0	42.9	0.0	T
	SO1	49.2	0.0	50.8	0.0	T
	SO1	98.2	1.8	0.0	0.0	T
	SO1	85.3	14.7	0.0	0.0	T
	SO1	73.3	26.7	0.0	0.0	T
	SO1	57.3	42.7	0.0	0.0	T
	SO1	50.2	49.8	0.0	0.0	T
	SO4	100.0	0.0	0.0	0.0	T
	SO4	98.8	0.0	0.0	1.2	T
	SO4	85.3	0.0	0.0	14.7	T
	SO4	75.2	0.0	0.0	24.8	T
	SO4	57.5	0.0	0.0	42.5	T
	SO4	50.1	0.0	0.0	49.9	T
	SO4	98.8	0.0	1.2	0.0	T
	SO4	92.7	0.0	7.3	0.0	T
	SO4	75.1	0.0	24.9	0.0	T
	SO4	65.0	0.0	35.0	0.0	T
	SO4	50.3	0.0	49.7	0.0	T
	SO4	98.3	1.7	0.0	0.0	T
	SO4	91.6	8.4	0.0	0.0	T
	SO4	75.1	24.9	0.0	0.0	T

	SO4	64.7	35.3	0.0	0.0	T
	SO4	49.9	50.1	0.0	0.0	T
**Pure adulterants**	FO	0.0	0.0	0.0	100.0	
	O3C	0.0	0.0	100.0	0.0	
	PO	0.0	100.0	0.0	0.0	

Table S3: Fatty acid composition (% of total fatty acids) in krill oil (KO), cod liver oil (CLO), salmon oil (SO), palm oil (PO), ω-3 concentrates in ethyl ester (O3C) and fish oil (FO).

Sample name	Major fatty acid content (% of total fatty acids)										
	C14:0	C16:0	C18:1 ω-9	C20:5 EPA	C22:5 DPA	C22:6 DHA	Total PUFA	Total MUFA	Total SFA	Total ω-3	EPA+DPA+DHA
**Krill oil (KO)**											
**K1**	10.6	23.4	11.6	20.5	0.2	9.8	37.2	27.1	35.8	35.4	30.5
**K2**	10.7	23.5	11.6	19.2	0.2	9.7	38.8	25.4	35.7	37.0	29.1
**K3**	11.1	23.5	12.3	19.5	0.2	8.0	36.5	27.5	36.1	34.2	27.7
**K4**	5.9	17.2	14.3	20.3	2.0	10.6	39.5	32.0	28.5	37.6	32.9
**K5**	11.3	24.2	13.0	19.9	0.2	8.4	34.0	29.0	37.1	32.6	28.5
**K6**	11.4	21.5	14.1	17.4	0.5	9.6	34.0	30.6	35.3	32.5	27.4
**Cod liver oil (CLO)**											
**CLO1**	4.5	10.9	16.9	8.0	0.8	10.6	23.6	58.4	18.0	22.0	19.4
**CLO2**	4.7	11.7	16.6	8.7	0.8	10.6	24.0	57.0	19.1	22.6	20.0
**CLO3**	5.2	12.8	17.3	10.1	0.9	10.1	24.7	54.4	20.9	23.2	21.1
**CLO4**	4.3	10.2	16.1	8.7	0.7	11.4	26.3	57.1	16.7	24.1	20.8
**CLO5**	4.4	10.4	16.2	8.6	0.7	11.2	25.7	57.4	16.9	23.6	20.5
**CLO6**	4.6	11.8	17.0	8.8	0.6	10.2	25.9	55.2	18.8	24.1	19.6
**Salmon oil (SO)**											
**SO1**	5.3	16.1	14.8	10.1	2.0	11.3	29.2	45.3	25.4	26.9	23.4
**SO2**	4.8	14.2	19.3	11.2	1.0	9.3	28.2	49.5	22.4	24.8	21.5
**SO3**	4.9	15.0	15.5	9.9	1.6	10.7	27.1	49.6	23.3	25.5	22.2
**SO4**	1.7	7.1	34.0	11.0	1.4	10.3	42.5	45.2	12.3	30.4	22.7
**SO5**	4.5	12.3	21.3	19.4	1.8	12.2	43.1	36.1	20.8	37.9	33.4
**SO6**	6.0	15.1	15.3	12.5	1.5	11.3	30.0	45.5	24.5	28.6	25.3
**Cheap oil as adulterants**											
**PO**	1.1	42.4	41.9	0.0	0.0	0.0	8.9	43.0	48.1	0.1	0.0
**O3C**	0.2	1.2	8.0	41.0	3.5	28.3	76.7	18.5	4.8	76.0	72.8

	C14:0	C16:0	C18:1 ω-9	C20:5 EPA	C22:5 DPA	C22:6 DHA	Total PUFA	Total MUFA	Total SFA	Total ω-3	EPA+DPA+DHA
**FO**	8.9	19.2	11.6	20.1	1.6	13.2	40.1	27.5	32.4	39.0	34.8
**Reference value from codex***											
**KO**	5.0 - 13.0	17.0 - 24.6	6.0 - 14.5	14.3 - 28.0	0.0 - 0.7	7.1 - 15.7	-	-	-	-	-
**CLO**	2.0 - 6.0	7.0 - 14.0	12.0 - 21.0	7.0 - 16.0	0.5 - 3.0	6.0 - 18.0	-	-	-	-	-
**SO wild**	2.0 - 5.0	10.0 - 16.0	8.0 - 16.0	6.5 - 11.5	1.5 - 3.0	6.0 - 14.0	-	-	-	-	-
**SO farmed**	1.5-5.5	6.5-12.0	30 – 47	2.0 – 6.0	1.0– 2.5	3.0 – 10.0	-	-	-	-	-

Abbreviations: EPA = eicosapentaenoic acid; DPA = docosapentaenoic acid; DHA = docosahexaenoic acid; SFA = saturated fatty acid; MUFA = mono-unsaturated fatty acid; PUFA = poly unsaturated fatty acid; CLO= cod liver oil; KO = krill oil; SO = salmon oil

*Reference value from codex standard of fish oil supplements (329-2017) by Food and Agriculture Organization of the United Nations (FAO) and world health organization (WHO)

Table S4: The model performance of PLSR for quantification of major fatty acids (FAs) content in krill oil, cod-liver oil and salmon oil. The *italicized* rows indicate the best performing model for each FAs type.

		**No. factors**	**Calibration**		**Cross-validation**		**Prediction (test set)**			
			**r^2**	**$RMSE_C$**	**r^2**	**$RMSE_{cv}$**	**r^2**	**slope**	**offset**	**$RMSE_P$**
**Total ω-3% (model range: 0 to 100 %)**	Raman	2	0.92	3.1%	0.92	3.3%	0.94	0.97	1.1	2.4%
	IR	2	0.89	3.8%	0.89	3.9%	0.95	0.88	3.4	2.3%
	Low-level fusion	*2*	*0.96*	*2.5%*	*0.95*	*2.6%*	*0.96*	*0.98*	*0.56*	*1.9%*
**EPA% (model range: 0 to 100 %)**	Raman	2	0.91	2.1%	0.90	2.2%	0.86	0.84	2.4	2.4%
	IR	*2*	*0.91*	*2.2%*	*0.90*	*2.2%*	*0.95*	*0.86*	*2.2*	*1.5%*
	Low-level fusion	2	0.95	1.6%	0.95	1.6%	0.94	0.90	1.6	1.7%
**DHA% (model range: 0 to 100 %)**	Raman	2	0.86	1.5%	0.85	1.6%	0.83	0.83	2.2	1.5%
	IR	*2*	*0.80*	*1.8%*	*0.78*	*1.8%*	*0.90*	*0.78*	*2.0*	*1.1%*
	Low-level fusion	*2*	*0.91*	*1.2%*	*0.91*	*1.2%*	*0.91*	*0.86*	*1.4*	*1.1%*
**EPA+DPA+DHA% (model range: 0 to 100 %)**	Raman	2	0.92	3.2%	0.91	3.3%	0.90	0.95	1.9	2.9%
	IR	*2*	*0.89*	*3.7%*	*0.89*	*3.8%*	*0.95*	*0.86*	*3.6*	*2.1%*
	Low-level fusion	*2*	*0.95*	*2.5%*	*0.95*	*2.6%*	*0.95*	*0.95*	*1.3*	*2.1%*
**Total PUFA% (model range: 0 to 100 %)**	Raman	*2*	*0.90*	*3.5%*	*0.89*	*3.6%*	*0.83*	*0.85*	*4.1*	*4.0%*
	IR	2	0.88	3.7%	0.88	3.8%	0.79	0.76	6.7	4.4%
	Low-level fusion	*2*	*0.94*	*2.7%*	*0.94*	*2.8%*	*0.83*	*0.86*	*3.6*	*4.0%*

**MUFA%** (model range: 0 to 100 %)	Raman	2	0.67	6.2%	0.66	6.3%	0.66	0.59	17.3	6.1%
	IR	*2*	*0.74*	*5.6%*	*0.74*	*5.7%*	*0.75*	*0.84*	*5.6*	*5.2%*
	Low-level fusion	*2*	*0.74*	*5.5%*	*0.74*	*5.6%*	*0.76*	*0.80*	*7.7*	*5.2%*
**SFA%** (model range: 0 to 100 %)	Raman	3	0.72	4.4%	0.71	4.5%	0.71	0.56	11.6	4.8%
	IR	3	0.73	4.2%	0.72	4.3%	0.78	0.74	8.4	4.3%
	Low-level fusion	*3*	*0.74*	*4.1%*	*0.72*	*4.2%*	*0.79*	*0.70*	*8.8*	*4.1%*

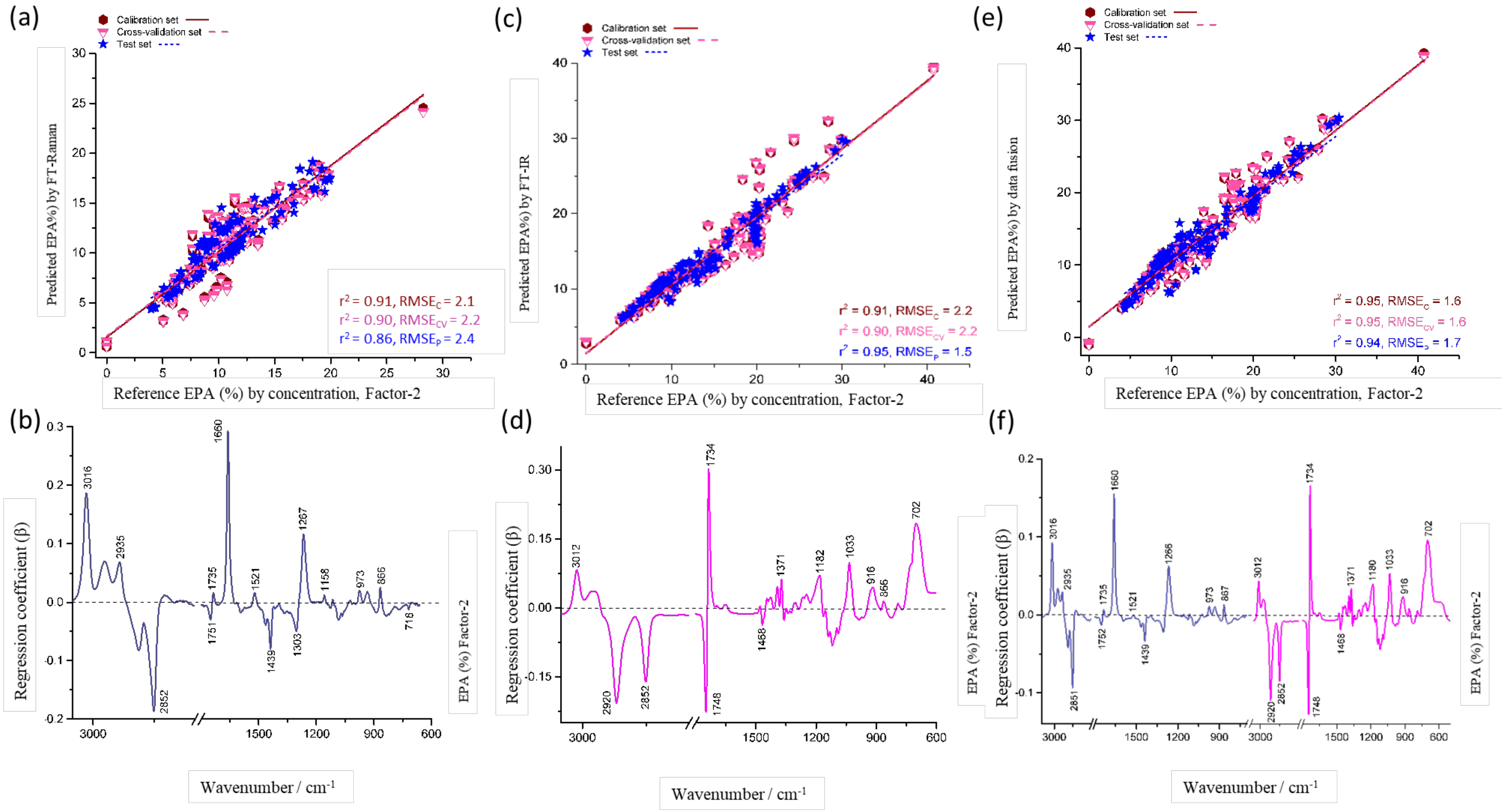

Figure S1: PLSR calibration lines and regression coefficients for quantitative prediction of EPA% concentration in marine oil (KO, CLO, SO) by Raman (a,b), IR (c,d) and low-level fused Raman plus IR data (e,f)

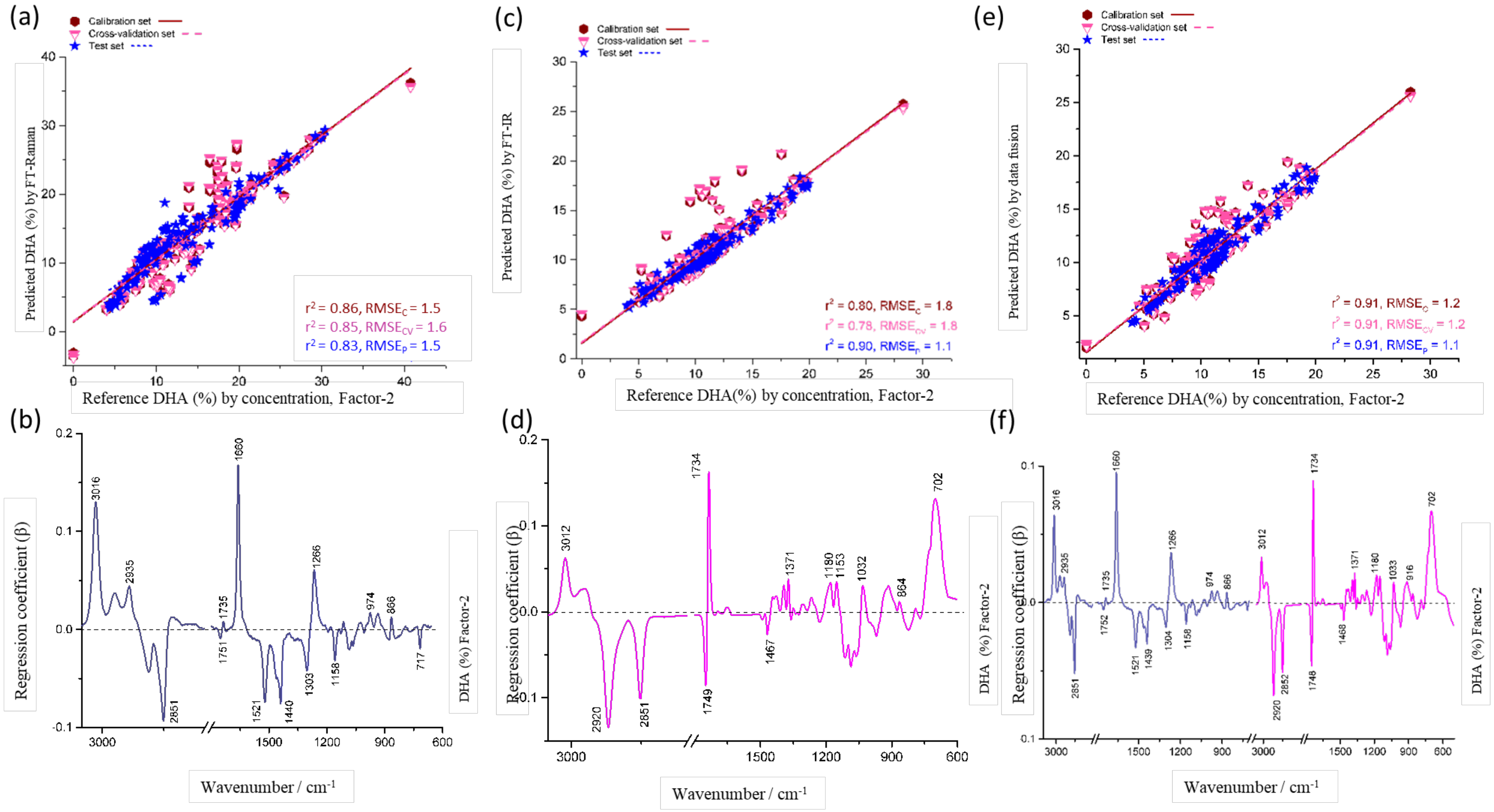

Figure S2: PLSR calibration lines and regression coefficients for quantitative prediction of DHA% concentration in marine oil (KO, CLO, SO) by Raman (a,b), IR (c,d) and low-level fused Raman plus IR data (e,f)

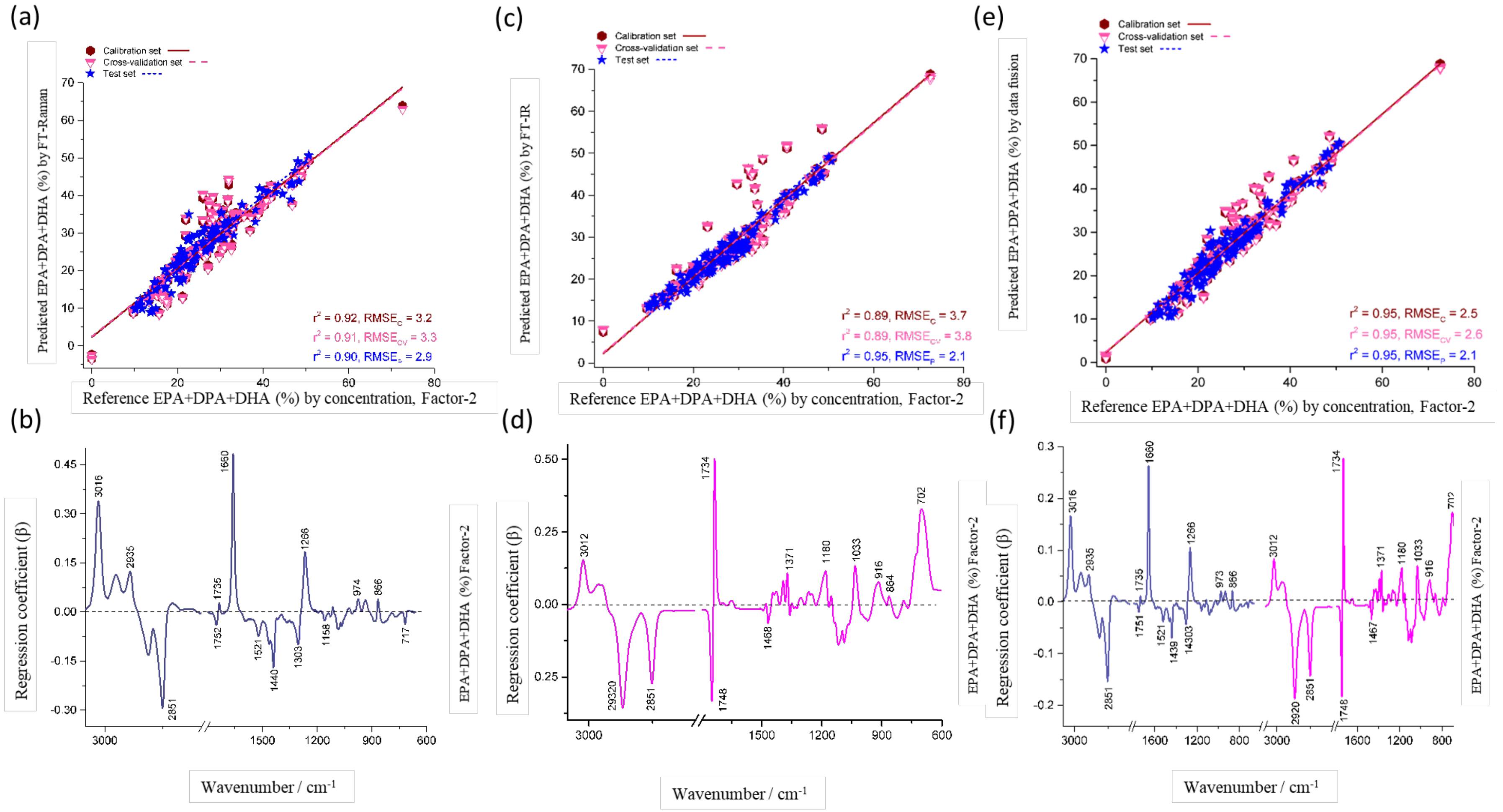

Figure S3: PLSR calibration lines and regression coefficients for quantitative prediction of EPA+DPA+DHA % concentration in marine oil (KO, CLO, SO) by Raman (a,b), IR (c,d) and low-level fused Raman plus IR data (e,f)

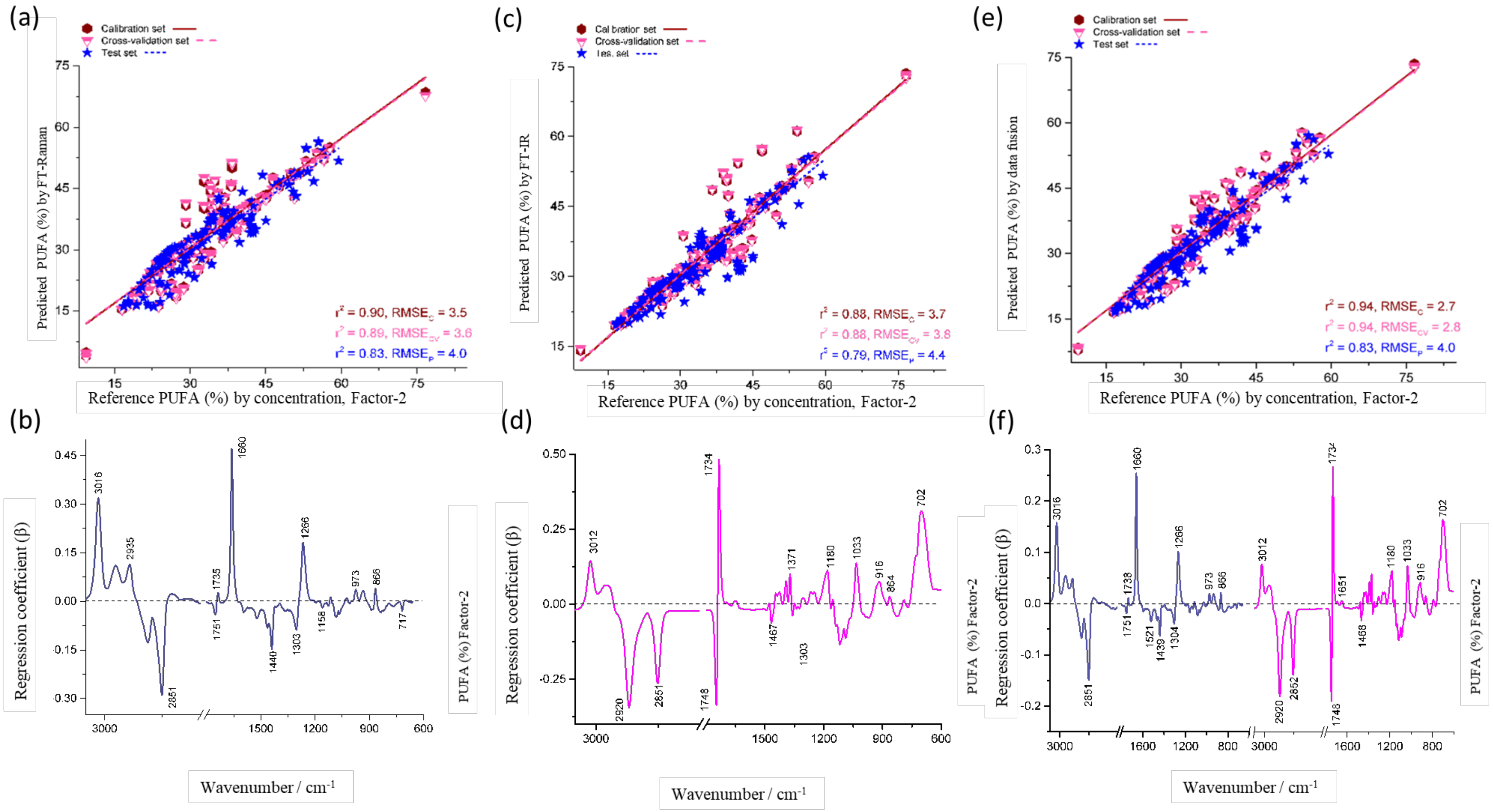

Figure S4: PLSR calibration lines and regression coefficients for quantitative prediction of PUFA % concentration in marine oil (KO, CLO, SO) by Raman (a,b), IR (c,d) and low-level fused Raman plus IR data (e,f)

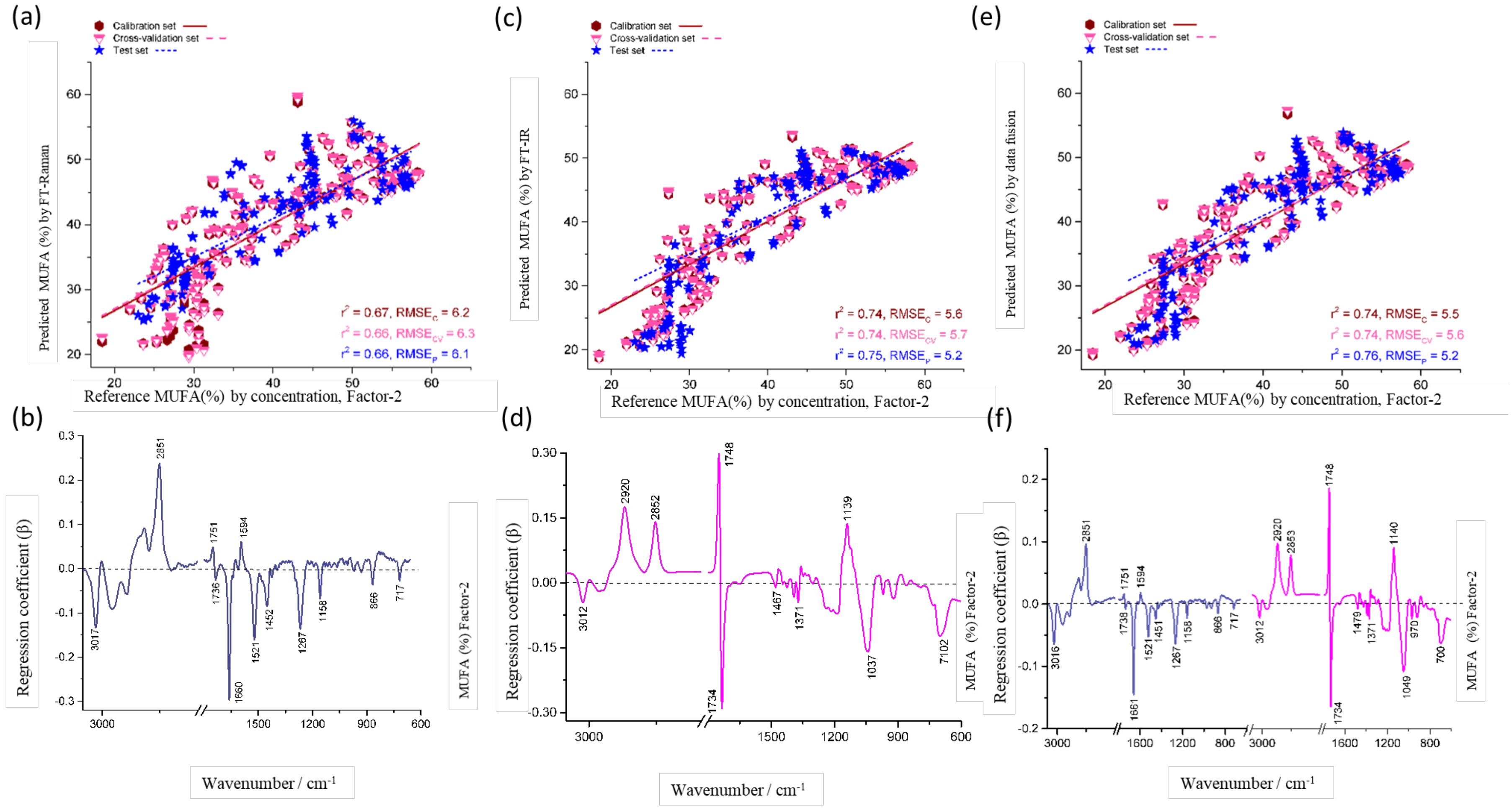

Figure S5: PLSR calibration lines and regression coefficients for quantitative prediction of MUFA % concentration in marine oil (KO, CLO, SO) by Raman (a,b), IR (c,d) and low-level fused Raman plus IR data (e,f)

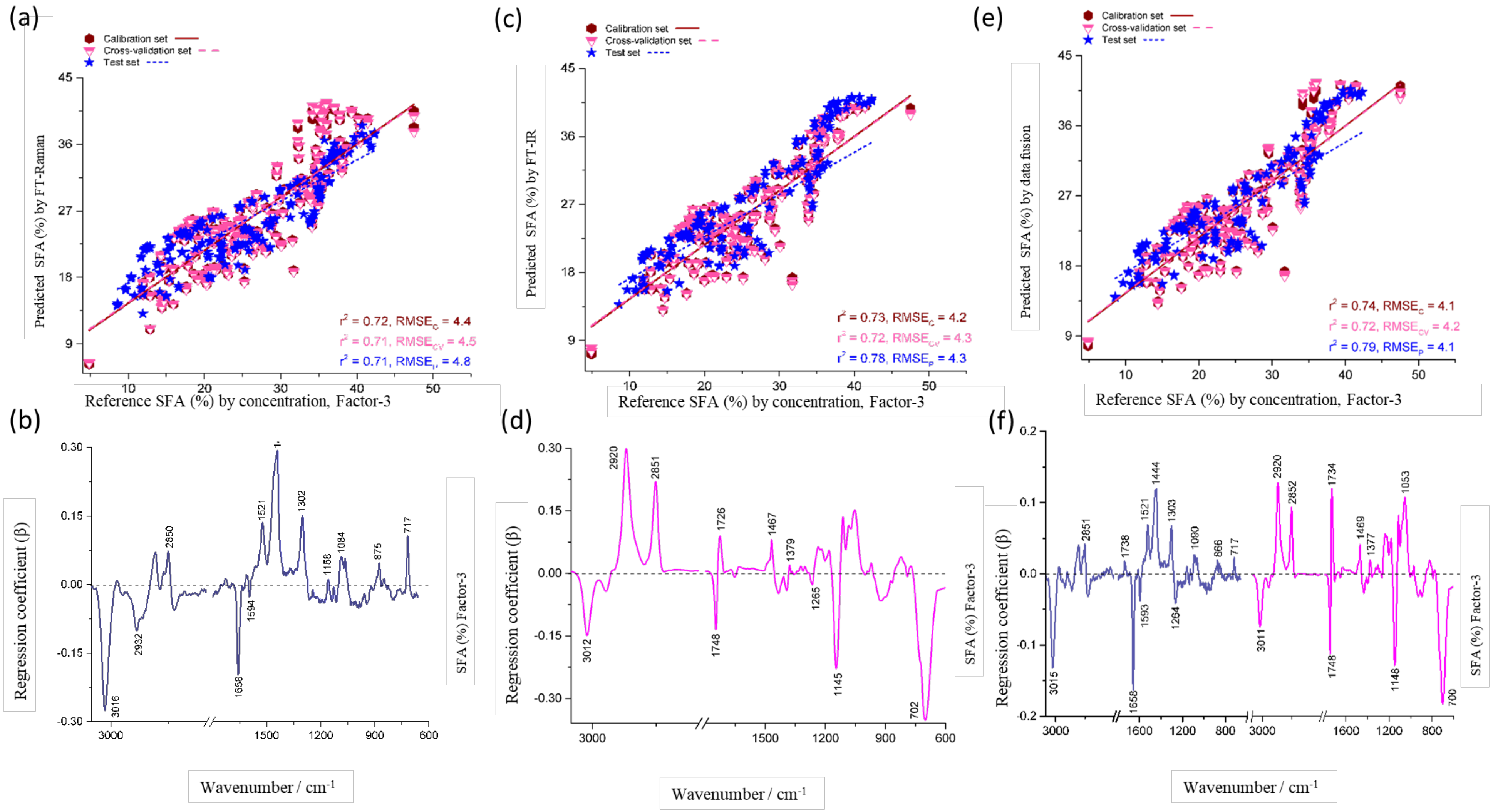

Figure S6: PLSR calibration lines and regression coefficients for quantitative prediction of SFA % concentration in marine oil (KO, CLO, SO) by Raman (a,b), IR (c,d) and low-level fused Raman plus IR data (e,f)
